# Exosomes secreted by stem cells from human exfoliated deciduous teeth contribute to functional recovery after traumatic brain injury by shifting microglia M1/M2 polarization in rats

**DOI:** 10.1186/s13287-017-0648-5

**Published:** 2017-09-29

**Authors:** Ye Li, Yuan-Yuan Yang, Jia-Li Ren, Feng Xu, Fa-Ming Chen, Ang Li

**Affiliations:** 1 Key Laboratory of Shaanxi Province for Craniofacial Precision Medicine Research, Xi’an, Shaanxi China; 2 Department of Periodontology, College of Stomatology, Xi’an Jiaotong University, Xi Wu Road No.98, Xi’an, Shaanxi 710004 China; 3 The Key Laboratory of Biomedical Information Engineering of Ministry of Education, Xi’an Jiaotong University School of Life Science and Technology, Xi’an, China; 4 Bioinspired Engineering and Biomechanics Center (BEBC), Xi’an Jiaotong University, Xi’an, China; 5 Department of Periodontology, School of Stomatology, Fourth Military Medical University, Xi’an, People’s Republic of China

**Keywords:** Traumatic brain injury, Stem cells from human exfoliated deciduous teeth, Exosomes, Microglia, Neuroinflammation

## Abstract

**Background:**

Traumatic brain injury (TBI) is one of the major causes of mortality and disability for all ages worldwide. Mesenchymal stem cells (MSCs)-originated exosomes have provided therapeutic effects. However, as an indispensable component of MSCs, whether odontogenic stem cell-generated exosomes could benefit TBI is still unclear. Thus we aimed to explore the potential of stem cells from human exfoliated deciduous teeth-originated exosomes (SHED-Ex) for the management of TBI.

**Methods:**

First, a transwell system was used to co-culture activated BV-2 microglia cells with SHED. The secretion levels of neuroinflammatory factors and nitrite were evaluated by enzyme-linked immunosorbent assay (ELISA) and Griess assay. Furthermore, purified SHED-Ex were co-cultured with activated BV-2. ELISA, Griess assay, flow cytometry, immunofluorescence, and qRT-PCR were performed to test the levels of inflammatory factors as well as the microglia phenotype. Finally, SHED and SHED-Ex were locally injected into TBI rat models. Basso, Beattie, and Bresnahan (BBB) scores were chosen to evaluate the motor functional recovery. Histopathology and immunofluorescence were performed to measure the lesion volume and neuroinflammation.

**Results:**

As a result, SHED-Ex could reduce neuroinflammation by shifting microglia polarization. The administration of SHED-Ex improves rat motor functional recovery and reduces cortical lesion compared with the control group 2 weeks post-injury (*P* < 0.05).

**Conclusions:**

The current study demonstrates for the first time that SHED-Ex contribute a therapeutic benefit to TBI in rats, at least in part by shifting microglia polarization to reduce neuroinflammation. The use of odontogenic stem cells, and indeed their exosomes, may be expanded for the treatment of TBI or other neurological disorders.

## Background

Stem cells from human exfoliated deciduous teeth (SHED) have been reported as an attractive source for tissue engineering and stem cell transplantation [1, 2]. With the representative characteristics of mesenchymal stem cells (MSCs), SHED display several advantages in the application of regenerative medicine. SHED are derived from dental pulps from young patients and hence represent a more immature population of postnatal stem cells with a higher proliferation rate [3]. Besides, SHED are more accessible for clinical application for noninvasive retrieval compared with MSCs from bone marrow, cartilage, muscle, and adipose tissue [4]. In addition, human exfoliated deciduous teeth are “disposable” tissues that are often taken as medical waste [5]. Therefore, fewer ethical concerns exist. With these advantages, SHED are recommended as a promising source of cell types for tissue engineering and have been utilized for the successful regeneration of a number of dental tissues in animal models. Interestingly, these cells also have the potential to give rise to non-dental cell lineages for use in the treatment of many systematic diseases, such as systemic lupus erythematosus, photoaging, and type 1 diabetes [6–9].

Defined as damage to the brain caused by external mechanical force, traumatic brain injury (TBI) has long been a major cause of mortality worldwide [10]. At present, few effective strategies or therapeutic interventions have appeared for treating TBI. Holding promise in the management of TBI, stem cell therapy has been shown to be an effective way for functional improvement [11]. However, it remains unclear whether SHED could benefit TBI as well. TBI is always followed by a cascade of events mediated by microglia, one of the main active cells of the central nervous system [12]. At the beginning, pro-inflammatory effects predominate, and a large number of inflammatory factors were released. Then, there is a transition of the microglia phenotype. They transit from the M1 polarization state to M2 to suppress pro-inflammatory mediators as well as enhance tissue repair [13]. However, chronic effects often occur because of ineffective inhibition of the pro-inflammatory process. Therefore, shifting microglia from M1 to M2 could be a target for the treatment of TBI. Given that neural stem cells (NSCs) benefit TBI patients by alternating the microglial polarization, we investigated here whether SHED could modulate the host microglia in a manner similar to that of NSCs.

Recently, increasing evidence has demonstrated that small-membrane vesicle exosomes with a size of approximately 30–100 nm in diameter play a pivotal role in stem cell therapy [14]. What is more, administrations of exosomes have demonstrated a therapeutic effect in multiple injury models [15–17]. It is believed that exosomes hold great potential as cell-free therapies because of their safety and low immunogenicity [18, 19]. Therefore, we hypothesize that exosomes derived from SHED (SHED-Ex) could be beneficial for TBI and thus provide a better way for treatments. Therefore, the present study primarily investigated whether SHED-Ex could benefit microglia polarization transition in vitro. More importantly, an experimental injury model was established with locally administered SHED-Ex to investigate motor functional recovery.

## Methods

### Culture of BV-2 microglia and TERT-SHED

Two immortalized cell lines were used in the present study. BV-2 was obtained from The Chinese Academy of Sciences, and TERT-immortalized SHED were resuscitated from TERT-SHED in our previous report [20]. Cells were conventionally cultivated with Dulbecco’s modified Eagle’s medium (DMEM, Gibco, Grand Island, NE, USA) containing 1% penicillin/streptomycin (Gibco) and 10% fetal bovine serum (FBS, Gibco) at 37 °C.

### Characterization of BV-2 microglia and TERT-SHED

The microglia phenotype of BV-2 was characterized by flow cytometry and immunofluorescence. Cells were stained with FITC-labeled CD11b and APC-labeled F4/80 antibodies and analyzed by BD FACSCalibur flow cytometer (BD Biosciences, San Jose, CA, USA). Rat anti-mouse CD11b, Iba-1, and F4/80 were used in the immunofluorescence, and the protocol was as previously described [21]. The results were analyzed by Olympus FSX100 fluorescence microscope (Olympus Corp., Tokyo, Japan). The characterization of TERT-SHED was performed previously [20].

### Microglia stimulation and ELISA

BV-2 were incubated with 1 μg/ml lipopolysaccharide (LPS) for 24 h to induce a pro-inflammatory phenotype. Enzyme-linked immunosorbent assay (ELISA) was performed to measure the concentrations of tumor necrosis factor alpha (TNF-α) and interleukin-6 (IL-6) in cell supernatants. The experiments were all performed in triplicate three times.

### Transwell assay

The co-culture experiment was performed by transwell assay as shown in Fig. 2a, the exchange of factors was allowed without cell-to-cell contact. In a transwell system, activated BV-2 were plated in the lower chamber, and TERT-SHED were plated in the upper chamber. Monocultures of activated BV-2 microglial cells were processed as controls.

### Exosome enrichment and characterization

TERT-SHED were cultured in DMEM with exosome-free FBS (SBI, Palo Alto, CA, USA) for 48 h. The cell culture media was then collected and mixed with the ExoQuick exosome isolation solution, and the following steps were performed in line with the manufacturer's instructions. PE-labeled CD81, CD63, and CD9 were used to evaluate the characteristics of the isolated exosomes by flow cytometry. For further validation of SHED-Exo, 10 μM GW4869 (Sigma‐Aldrich, St Louis, MO,, USA) was used to inhibit exosomes release from SHED. Equal amounts of protein were extracted from SHED-Exo, SHED, and exosomes from GW4869-treated SHED for western blotting to detect the expression of CD9 and CD63.

### Electron microscopy

The enriched exosomes were first loaded onto a carbon-coated grid, and remained for 7 min. With the excess fluid removed, samples were then stained in 3% phosphotungstic acid (pH 6.9) for 5 min. Transmission electron microscopy (TEM) was used for the analysis.

### Exosome labeling and internalization

Purified exosomes were labeled with CM-DiI (red) as previously described [22]. BV-2 microglia were seeded in 24-well plates for overnight attachment. The labeled exosomes were added the next day for a 48 h incubation. The nuclei were stained with DAPI before image acquisition.

### Griess assay

As the supernatant accumulation of nitrite is an indicator of the production of nitric oxide (NO), Griess assay was performed to determine the nitrite concentration. BV-2 microglia cells were activated as previously described, and different concentrations with equal volume (50, 100, 200, 400 μg/ml) of SHED exosomes were added into the culture medium. We incubated 50 μl of each supernatant with 50 μl Griess reagent (Promega, Madison, WI, USA) for 8 min. The absorbance was then measured at 520 nm. With a standard curve as the reference, the nitrite concentration was calculated.

### Flow cytometry and immunofluorescence

BV-2 microglia cells were activated as previously described, and different concentrations (50, 100, 200, 400 μg/ml) of SHED exosomes were added into the culture medium. Rat anti-mouse CD11b and CD68 were used to evaluate the M1 phenotype of the microglia. The changes were assessed by flowjo3.4.1 (FlowJo, LLC, Ashland, OR, USA). For immunofluorescence, confocal microscopy was used to analyze the images.

### qRT-PCR

Total RNA was extracted, cDNA was synthetized and real-time PCR was performed. Primers used are shown in Table 1. GAPDH was used as the reference. ABI 7500 Sequence Detection System (Applied Biosystems, Foster City, CA, USA) was used to analyze the results).

**Table 1:** Primer sequences

	Gene	Gene bank accession	Primer sequence (5′—3′) forward primer reverse primer	Product (bp)
Reference	β*-actin*	NM_007393	GCCCTGAGGCTCTTTTCCAG	51
			TGCCACAGGATTCCATACCC	
M1 phenotype	*CD11b*	NM_001082960.1	GAGCAGCACTGAGATCCTGTTTAA	64
			ATACGACTCCTGCCCTGGAA	
	*CD86*	NM_019388.3	ACGATGGACCCCAGATGCACCA	88
			GCGTCTCCACGGAAACAGCA	
	*CD16*	XM_006496658.2	TGTGTGTCGTCGTAGACGGT	396
			TTCGCACATCAGTGTCACCA	
	*MHCII*	NM_010382.2	ACAGGAATTGTGTCCACGGG	472
			AAGGCCTGGGTCAGGGATAA	
	*iNOS*	NM_010927.3	GACGAGACGGATAGGCAGAG	80
			GTGGGGTTGTTGCTGAACTT	
M2 phenotype	*CD206*	NM_000237.2	TCAGCTATTGGACGCGAGGCA	105
			TCCGGGTTGCAAGTTGCCGT	
	*IL-10*	NM_010548.2	CGGCTGAGGCGCTGT	51
			TGCCTTGCTCTTATTTTCACAGG	
	*Arg-1*	NM_007482.3	AGCCAATGAAGAGCTGGCTGGT	91
			AACTGCCAGACTGTGGTCTCCA	

### Animal model

A well-established rat TBI model was utilized in the present study via the “free-falling method” [23]. Adult male Wistar rats weighing 220–240 g were first anesthetized with chloral hydrate intraperitoneally. A stereotactic frame was used to induce the injury. A 2 mm diameter circular window was drilled 1 mm adjacent to the left side of anterior fontanelle, which corresponded to the cortical motor functional area. A 20 g weight was used to hit the injured area. The craniotomy was then closed. The impact of this injury is nearly equal to a moderate TBI in humans.

### Assessment of neurological function

Basso, Beattie, and Bresnahan (BBB) scores were used to assess the neurological motor function before TBI and on the 1st, 3rd, 7th, 14th and 21st day after TBI. The assessment was performed by three investigators blinded to the experimental groups [24]. BBB is a complex behavioral test with a scoring range of 0–21 that evaluates long-term functional recovery. The stages are divided into the early phase, intermediate phase, and late phase of recovery (score from 0–7, 8–13, 14–21 respectively).

### Tissue preparation, H&E staining, and immunofluorescent staining

After anesthesia, the brains were extracted and fixed by 4% paraformaldehyde, and then tissues were dehydrated in the 15% sucrose solution followed by a 30% sucrose solution. OCT-embedded tissues are ready for staining. H&E staining of the tissues was performed as previously reported [25]. Immunostaining was performed to identify CD68-positive cells in the lesion zone.

### Statistical analysis

Student’s *t* test and one-way ANOVA were used for the evaluation of the differences among groups. A *P* value less than 0.05 was considered as the significant difference.

## Results

### BV-2 cell line displays a normal microglia phenotype

**Fig. 1:**
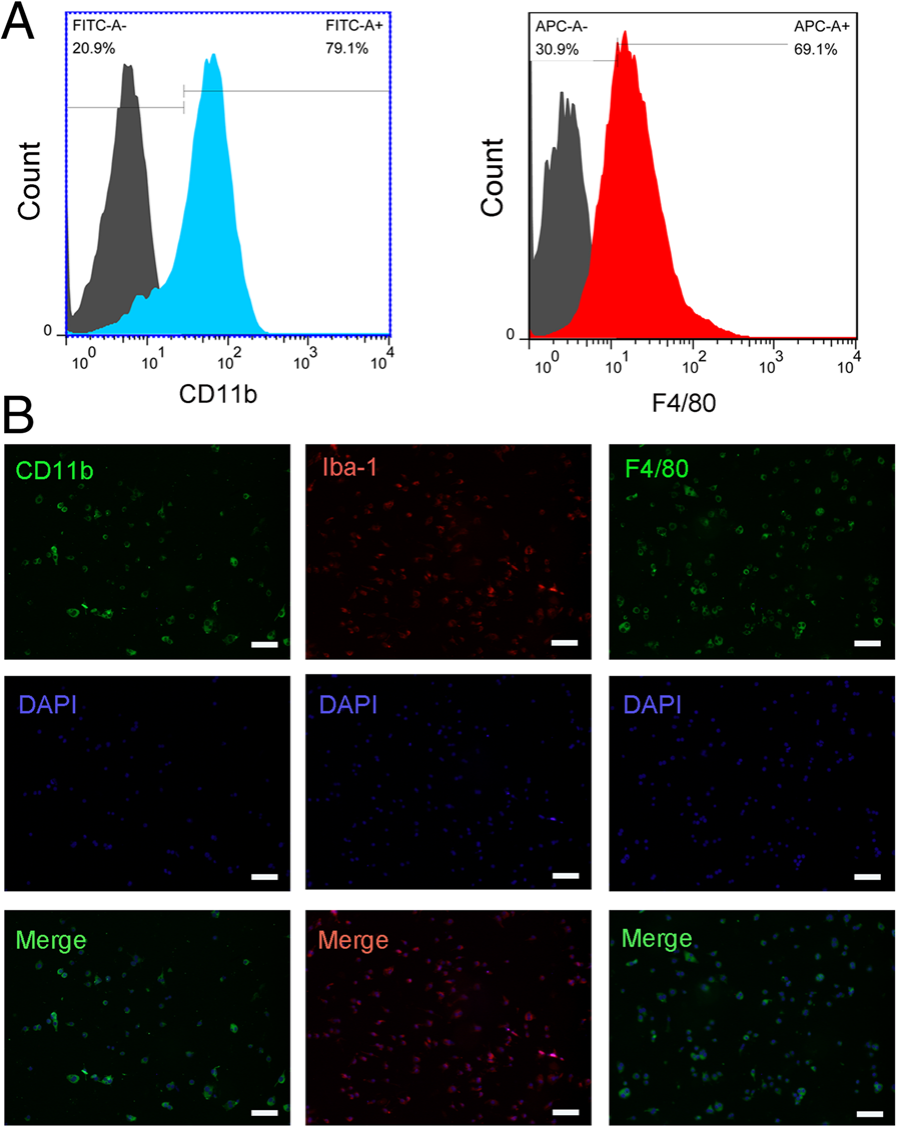
Phenotyping of BV-2 microglia. **a** Flow cytometry histograms showed the expression levels of surface markers: CD11b (FITC, *blue peaks*) and F4/80 (APC, *red peaks*); the numbers of positive cells are indicated at the *top right corner*. **b** Representative images of BV-2 microglia with respect to immunofluorescence observation showing positive expression of CD11b, Iba-1, and F4/80. Scale bar = 100 μm

To characterize the phenotype of the BV-2 cell line, we first evaluated the expression levels of classical markers that are used to identify microglia: CD11b, Iba-1, and F4/80. First, microglia surface markers CD11b and F4/80 were analyzed by flow cytometry. Both the markers detected high expression levels (Fig. 1a); isotype controls accounting for nonspecific binding are shown in gray. The expression of CD11b, Iba-1, and F4/80 were further confirmed by fluorescence microscopy (Fig. 1b); all three markers were strongly detected in BV-2 cells. These combined data suggest that BV-2 cells formed a pure population with biomarkers associated with normal microglia.

### The immunomodulatory effect on SHED co-cultured microglia

**Fig. 2:**
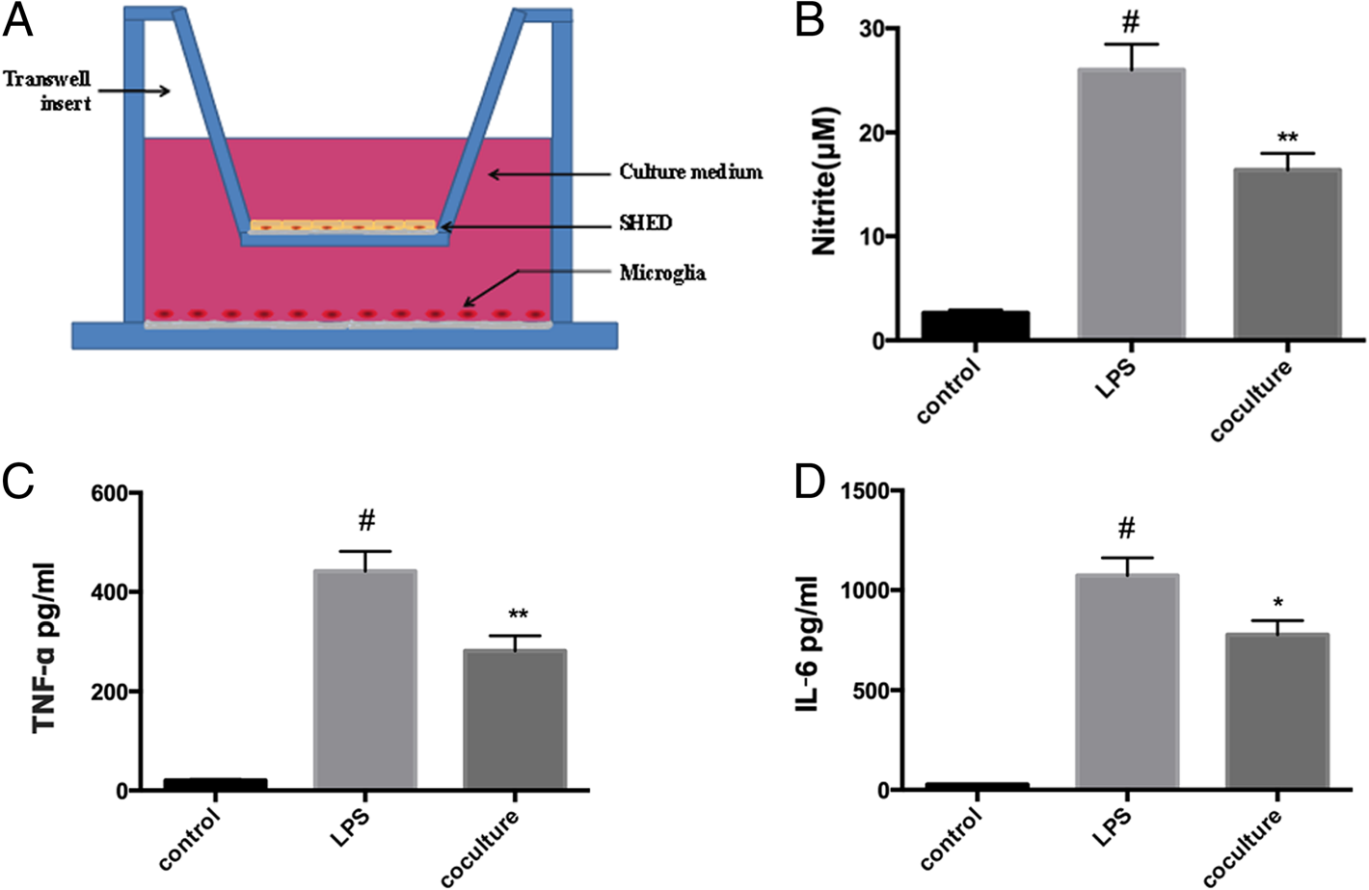
The immunomodulatory effect of SHED on BV-2 microglia. **a** The co-culture system of SHED and BV-2 microglia. In a transwell system, SHED and BV-2 microglia were cultured together, as shown. **b** Inhibitory effect of SHED on the production of nitrite. BV-2 were treated with 1 μg/ml LPS for 24 h and then followed by SHED co-culture. The content of nitrite was measured by Griess assay. **c**, **d** The effect of SHED on pro-inflammatory cytokines TNF-α and IL-6. An ELISA assay was performed. ^#^ *P* < 0.05 compared with nonactivated microglia cells. ^*^ *P* < 0.05, ^**^ *P* < 0.01 compared with LPS-only treated cells. *IL-6* interleukin-6, *LPS* lipopolysaccharide, *SHED* stem cells from human exfoliated deciduous teeth, *TNF-*α tumor necrosis factor alpha

A transwell co-culturing system was used to evaluate the effect of SHED on BV-2 microglia (Fig. 2a). A Griess assay and ELISA test were used to determine the levels of nitrite and of inflammatory factors TNF-α and IL-6 that are secreted by microglia. Nitrite secreted by activated microglia after co-culturing for 48 h were greatly decreased compared with the single activated microglia group (Fig. 2b). TNF-α and IL-6 showed a similar tendency to decrease (Fig. 2c, d).

### Identification of SHED-Ex

**Fig. 3:**
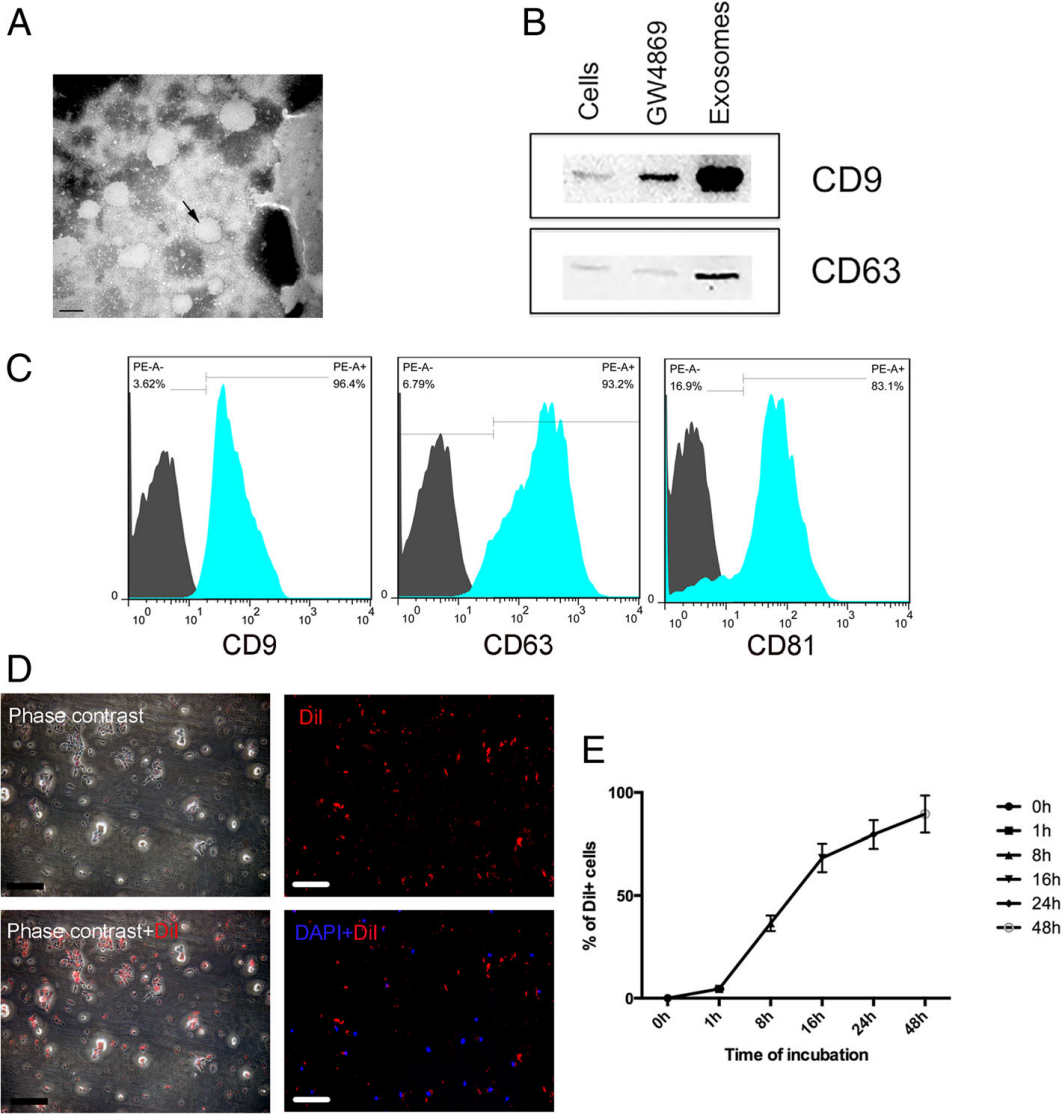
Characterization of SHED-Ex and internalization assay. **a** Morphologic observation of SHED-Ex by transmission electron microscopy (scale bar = 50 nm). **b** Surface exosomal markers (CD9, CD63) were analyzed using Western blotting in SHED, exosomes from GW4869-treated SHED, and SHED-Exo. **c** Flow cytometric analysis showing the presence of CD9, CD63, and CD81 in exosomes derived from SHED. **d** Immunofluorescence image showing the internalization of CM-Dil-labeled exosomes (*red*) by microglia (scale bar = 150 μm). **e** DiI uptake was analyzed by flow cytometry with different incubation times

When the purified exosomes generated from SHED were observed by TEM, most of the exosomes had a characteristic morphology with a size ranging from 30 to 100 nm (Fig. 3a). Then the expression of surface exosomal markers CD9 and CD63 were confirmed by western blotting (Fig. 3b). Flow cytometry showed that surface exosomal markers such as CD9, CD63, and CD81 were highly expressed (Fig. 3c). Moreover, the internalization of the exosomes was tested. The exosomes were first labeled with CM-DiI and were then added into the BV-2 microglia culture system for incubation. After 48 h we could detect strong red fluorescence in the microglia cells (Fig. 3d) and the percentage of DiI cells increased along with the incubation time (Fig. 3e).

### SHED-Ex display a robust response to pro-inflammatory stimuli

**Fig. 4:**
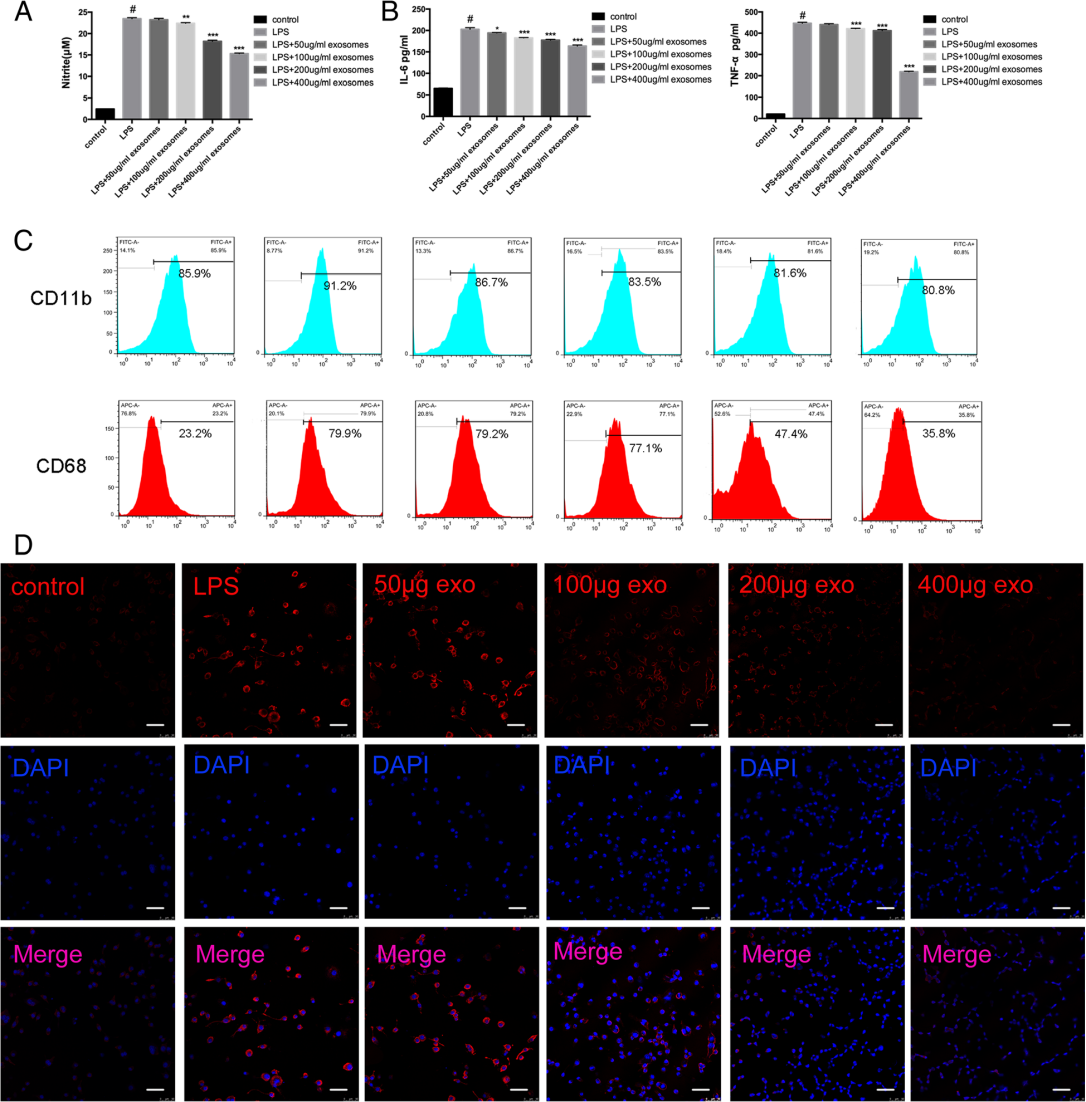
SHED-Ex displays a robust response to pro-inflammatory stimuli. **a** Griess assay for the microglia production of nitrite with different concentrations of SHED-Ex. **b** ELISA assay for pro-inflammatory cytokines TNF-α and IL-6 with different concentrations of SHED-Ex. **c** Flow cytometry for the expression levels of CD11b and CD68 of microglia with different concentrations of SHED-Ex. **d** Immunofluorescence staining and confocal analysis for CD68. ^#^ *P* < 0.05 compared with non-activated microglia cells. ^*^ *P* < 0.05, ^**^ *P* < 0.01, ^***^ *P* < 0.001 compared with LPS-only treated cells. *IL-6* interleukin-6, *LPS* lipopolysaccharide, *TNF-*α tumor necrosis factor alpha

To determine the effects of SHED-Ex on microglia, a Griess assay, an ELISA test, flow cytometry and immunofluorescence were used. Activated microglia were treated with different concentrations of SHED-Ex (50, 100, 200, 400 μg/ml) for 48 h. We sought to determine whether SHED-Ex could affect microglia in response to the pro-inflammatory process. The results of the Griess assay demonstrated that SHED-Ex decreased the nitrite concentration in a dose-dependent manner (Fig. 4a). What is more, an ELISA test was used to measure the concentrations of TNF-α and IL-6 in the microglia supernatants. Both the cytokines were significantly decreased in a dose-dependent manner in the SHED-Ex group compared with the single LPS group (Fig. 4b). The expression levels of CD11b and CD68 were next evaluated to measure the pro-inflammatory phenotype. The flow cytometry results showed that SHED-Ex strongly decreased the expression level of CD68 in a dose-dependent manner, while the influence in CD11b is not obvious (Fig. 4c). In accordance with the flow cytometry results, the immunofluorescence results demonstrated that SHED-Ex significantly affected the expression level of CD68 (Fig. 4d). The combined results suggest that SHED-Ex could display a robust response to pro-inflammatory stimuli in a dose-dependent manner.

### Microglial M1 phenotype polarization was prevented by SHED-Ex while M2 phenotype was promoted

**Fig. 5:**
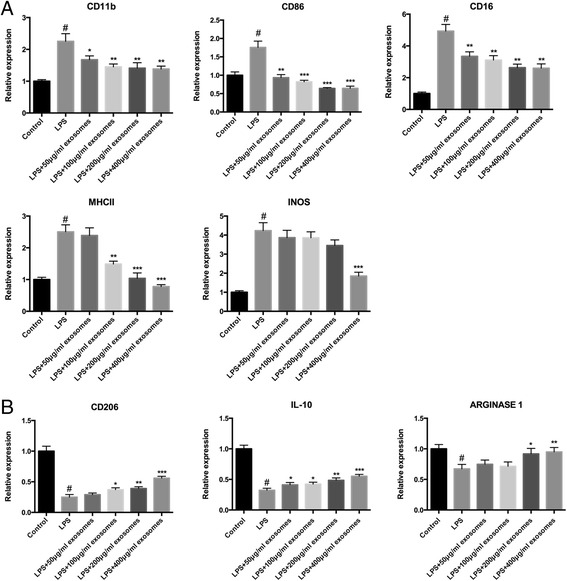
Detection of mRNA expression of M1 and M2 phenotypic markers of microglia. **a** M1-associated markers CD11b, CD86, CD16, MHC II, and iNOS were significantly decreased after exosome treatment compared with the single LPS stimulation group. **b** M2-associated markers CD206, IL-10, and ARGINASE 1 were significantly increased after exosome treatment compared to the single LPS stimulation group. ^#^ *P* < 0.05 compared with non-activated microglia cells. ^*^ *P* < 0.05, ^**^ *P* < 0.01, ^***^ *P* < 0.001 compared with LPS-only treated cells. *IL-10* interleukin-10, *LPS* lipopolysaccharide

Microglia M1/M2-associated markers were detected via mRNA levels. The data showed that the mRNA levels of M1 polarization-associated markers CD11b, CD86, CD16, MHCII, and iNOS were all significantly decreased in BV-2 microglia (Fig. 5a). While the mRNA levels of M2 polarization-associated markers CD206, IL-10 and ARGINASE 1 were all significantly increased in BV-2 microglia (Fig. 5b) compared with single LPS treatment.

### Administration of SHED-Ex significantly promotes functional motor recovery in rats after TBI

**Fig. 6:**
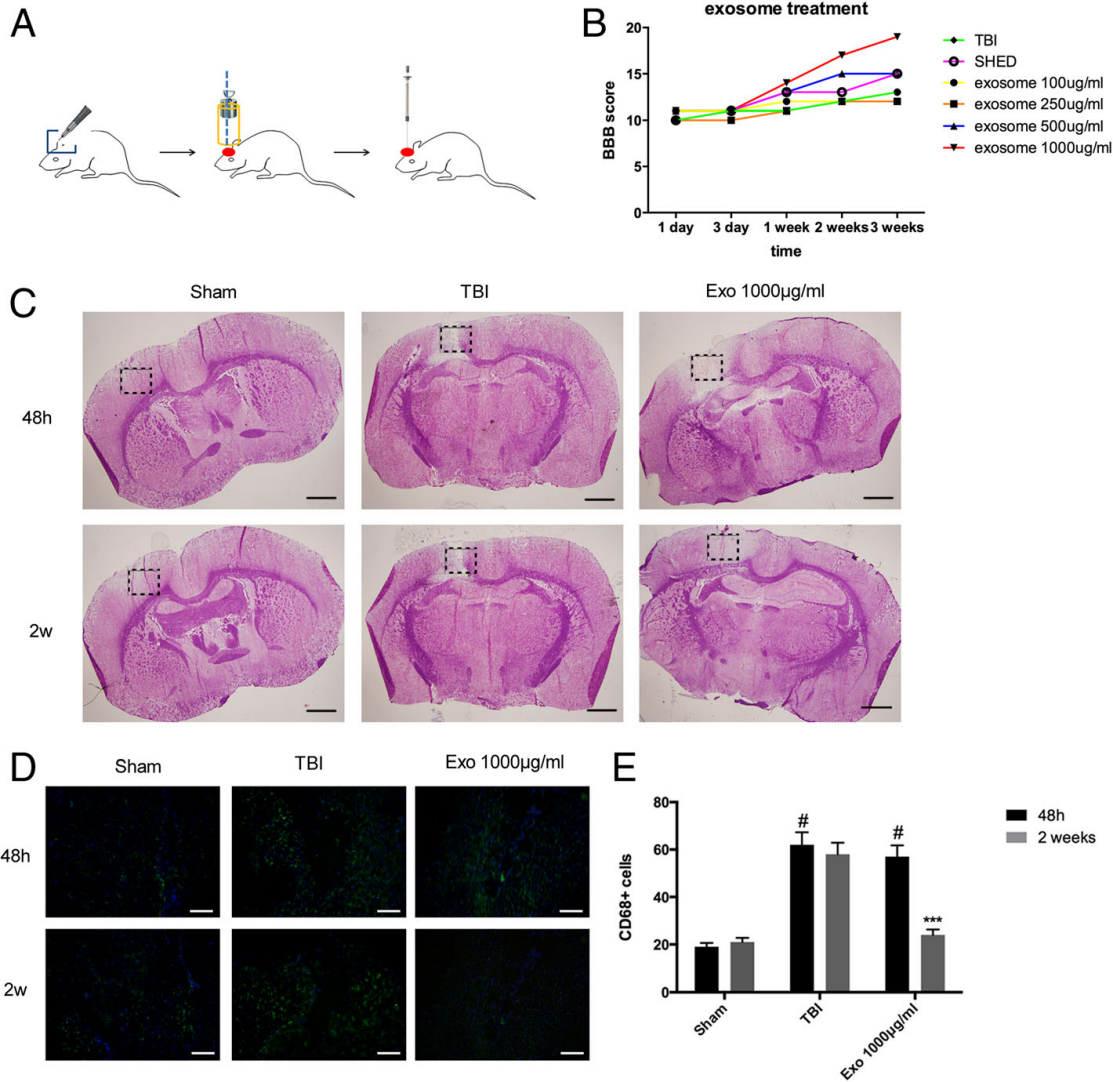
SHED-Ex promotes functional motor recovery after TBI. **a** Establishment of the TBI model in rats. **b** Motor assessment. The Basso, Beattie, and Bresnahan (BBB) scores for each group from 1 to 21 days after TBI. **c** Lesion reconstruction at 48 h and 2 weeks post-transplant. Scale bar = 50 μm (**d**) CD68 staining for activated microglia at 48 h and 2 weeks post-transplant. Scale bar = 50 μm. (**e**) Treatment with SHED-Ex significantly reduces the number of CD68+ microglia in the brains of rats after TBI. ^#^ *P* < 0.05 compared with sham group. ^***^ *P* < 0.001 compared with TBI group. *SHED-Ex* stem cells from human exfoliated deciduous teeth-originated exosomes, *TBI* traumatic brain injury

Functional motor measurement was performed in rats using standardized BBB scores. The recovery of motor function is graded on a scale of 0–21; the higher the score, the better the recovery. The BBB score was 10-12 in rats with TBI (all the groups) on day 1 post-TBI, indicating that motor deficits in all rats were comparable at the beginning. A significant promotion was found over time in the SHED-treated and SHED-Ex-treated animals from day 3–21 compared with day 1 post-injury, suggesting that there is a significant spontaneous recovery in treatment groups after TBI. More importantly, compared with the SHED group, functional motor recovery was significantly increased in the 1000 μg/ml SHED-Ex group (Fig. 6b). The cortical lesion volume was measured in the different groups at 48 h and 2 weeks post-TBI (Fig. 6c). Significant recovery was observed between the single TBI group and the 1000 μg/ml SHED-Ex group. Microglia were identified with CD68 immunofluorescence staining in the brain after TBI. As shown in Fig. 6d, the single TBI group demonstrated an increased density of CD68+ cells compared with the sham group. SHED-Ex could reduce the CD68+ cell density. From the statistical data, SHED-Ex treatment significantly inhibits CD68+ cells in rat brain (Fig. 6e).

## Discussion

TBI is one of the leading causes of severe disability and mortality for all ages worldwide. Affected patients are often accompanied by resultant motor or cognitive dysfunction, leaving devastating effects on the ability to continue with a normal life. As a potential treatment strategy, stem cell therapy has received much attention over the years. Neural stem cells are perhaps the fundamental choice for transplantation, but the availability is somehow limited, and there are not always enough cells for therapy [26, 27]. Interestingly, mesenchymal stem cells from bone marrow, adipose tissue, and umbilical cords showed an attractive therapeutic effect by improving the impaired function in animal models [28–30]. However, as one of the important components of MSCs, it remains unclear whether odontogenic stem cells could benefit TBI as well. Until now, with mesenchymal stem cell characteristics, odontogenic stem cells that have been well characterized are as follows: dental follicle progenitor cells (DFPCs), dental pulp stem cells (DPSCs), stem cells from apical papilla (SCAP), periodontal ligament stem cells (PDLSCs), and stem cells from exfoliated deciduous teeth (SHED) [31]. Among them, SHED strongly express NANOG, SOX-2, and OCT-4, all of which are embryonic stem cell markers, and have been reported to exhibit outstanding craniomaxillofacial tissue regeneration capacities [32]. The current study was designed as a first attempt to use SHED for the administration in TBI.

In our present in vitro study, the concentration of inflammatory factors TNF-α and IL-6 was evaluated with or without SHED treatment. It was found that SHED could significantly reduce the secretion of inflammatory factors by activated microglia. Previously, the administration of bone marrow mesenchymal stem cells in a TBI rat model also demonstrated the anti-inflammatory functions, which is in line with our results [33]. Microglia have been reported as the most important resident immune cells in the central nervous system [34]. After TBI, microglia were soon activated to release pro-inflammatory factors, for example, IL-6 and TNF-α, which may in turn exacerbate brain damage. The reduced release of these inflammatory factors by SHED treatment may be beneficial in TBI by inhibiting neuroinflammation. Nitrite is the end product of NO, which plays neurotoxic roles after TBI. Therefore, the decreased nitrite concentration in the SHED co-culture group may also account for the protective effects found in TBI.

The present study demonstrated that after co-culturing SHED with microglia for 48 h in the transwell system, the concentration of nitrite and inflammatory factors IL-6 and TNF-α all decreased compared with the single activated microglia group. As is reported previously, MSCs could secrete anti-inflammatory growth factors [35, 36]. While increasing data suggest that exosomes derived from mesenchymal stem cells was an also an important player in repairing tissue damage [37]. Therefore, we hypothesize that exosomes produced by the co-cultured SHED (SHED-Ex) may contribute the decreased secretion of inflammatory factors by microglia. To clarity if and to what extent SHED-Ex influence the co-cultured microglia, we designed the following experiments. We found that purified SHED-Ex was alone able to alter the polarization of microglia, and inhibit the inflammatory effects of M1 microglia. Therefore, we concluded that SHED-Ex, independent of the anti-inflammatory growth factors, could reduce inflammation, to an even larger extent than the reduction effect that was found in co-culture systems. It is reported that exosomes transport RNAs, proteins, and lipids to the targeted cells to reprogram cell behaviors [38]. Among them, noncoding RNAs, such as miRNAs or LncRNAs, have been reported to play crucial roles. Therefore, further studies are warranted to focus on specific noncoding RNAs that dominate the process. Once the specific molecule is revealed, the therapeutic efficiency will be enhanced by selective manipulation of the expression.

It has been demonstrated that microglia-mediated neuroinflammation plays a critical role in secondary brain injury in TBI [13]. In the present study, SHED-Ex could significantly reduce the pro-inflammatory microglia M1 phenotype cell markers; more importantly, it could do so in a dose-dependent manner. To further certify the role of SHED-Ex on microglial polarization, the mRNA levels of microglia M1/M2 phenotype markers were detected. As we hypothesized, after incubation with SHED-Ex for 48 h, a group of microglia was polarized from the pro-inflammatory phenotype into the anti-inflammatory phenotype, which led to a welcoming restoration after neuroinflammation. In this case, SHED-Ex were further investigated to detect the neuroprotective roles they may have played when administered to rats with TBI.

In the current study, 500 μg/ml SHED-Ex injected into rat brain rescued the cortical damage and improved the motor deficits resulting from TBI in rats. Actually, it was almost equivalent to the effect that occurred with a 10^5^/3 μl SHED treatment for TBI, while 1000 μg/ml exosomes provided a better functional recovery. Therefore, the dose-response efficacy was determined. However, whether a higher dose could provide better efficacy is unknown. Additionally, CD68+ microglia were used to identify activated M1 microglia after TBI. Compared with the TBI group, 1000 μg/ml SHED-Ex could significantly suppress the CD68+ microglia, which suggested the potential therapeutic mechanisms of SHED-Ex transplantations. In addition, we cannot ignore the possibility of how SHED-Ex may also act, such as the benefits of direct or indirect neurovascular regeneration. Future investigations are therefore planned to determine whether neurovascular regeneration could also be manipulated by SHED-Ex.

**Fig. 7:**
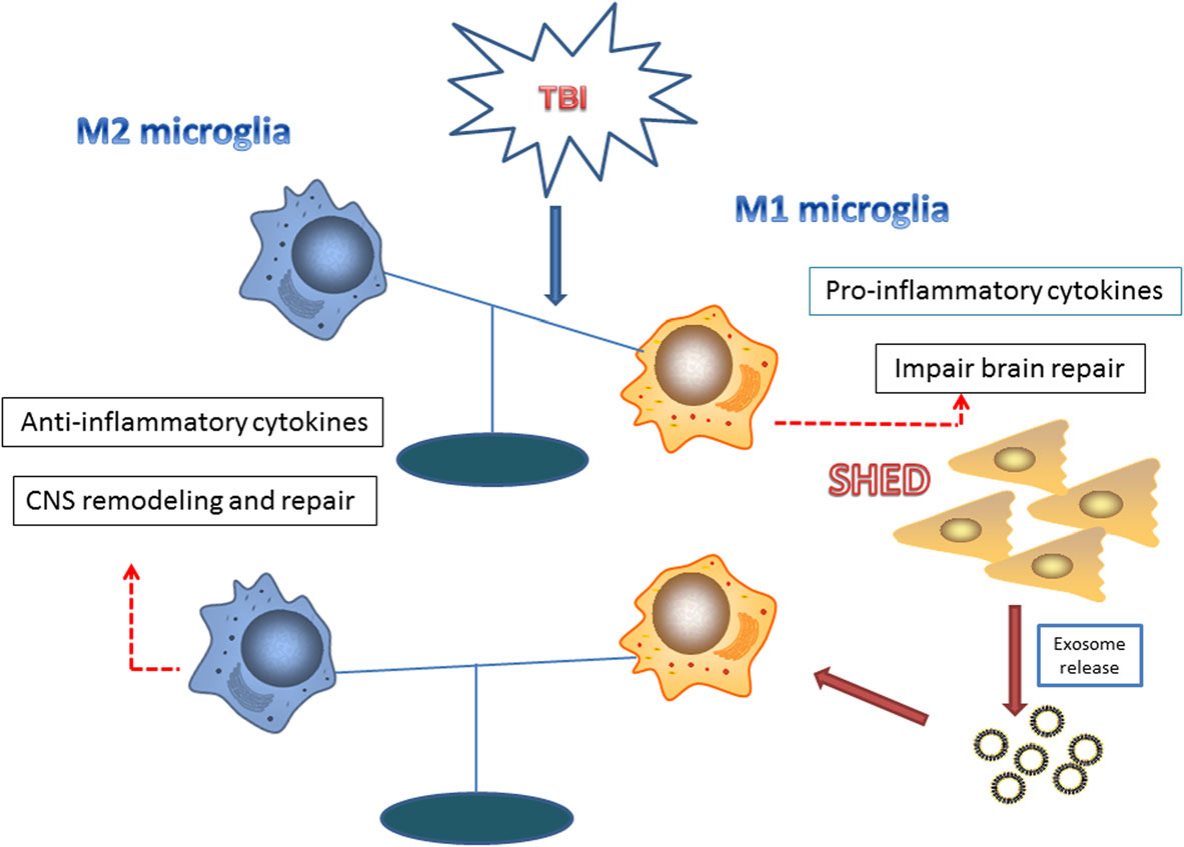
Schematic of SHED-Ex repair of impaired CNS: SHED-Ex shifts microglia M1/M2 polarization. SHED-Ex contact with activated microglia to promote robust M2 polarization. As a result, anti-inflammatory cytokines were released to repair. *CNS* central nervous system, *SHED-Ex* stem cells from human exfoliated deciduous teeth-originated exosomes, *TBI* traumatic brain injury

We combined the above data to explain the therapeutic mechanism of SHED-Ex. At the very beginning of TBI, microglia become more polarized towards M1 activation states over M2. By releasing pro-inflammatory cytokines as well as free radicals, M1 microglia-mediated chronic neuroinflammation exacerbated neurological impairments. In contrast, when SHED-Ex were added, M2 microglia were robustly activated, and thus the neuroinflammation was suppressed by anti-inflammatory cytokines. Long-term functional recovery and reduced neurodegeneration are therefore developed (Fig. 7).

## Conclusions

The current study demonstrated for the first time that the administration of SHED-Ex improves rat motor functional recovery and reduces neuroinflammation after TBI by shifting microglia M1/M2 polarization. This result may provide a novel therapeutic approach for the management of TBI and probably other neurological disorders.

## Abbreviations

BBB: Basso, Beattie, and Bresnahan
DFPCs: Dental follicle progenitor cells
DMEM: Dulbecco’s modified Eagle’s medium
DPSCs: Dental pulp stem cells
ELISA: Enzyme-linked immunosorbent assay
FBS: Fetal bovine serum
IL-6: Interleukin-6
LPS: Lipopolysaccharide
MSCs: Mesenchymal stem cells
NSCs: Neural stem cells
PDLSCs: Periodontal ligament stem cells
SCAP: Stem cells from apical papilla
SHED: Stem cells from human exfoliated deciduous teeth
SHED-Ex: Stem cells from human exfoliated deciduous teeth-originated exosomes
TBI: Traumatic brain injury
TNF-α: Tumor necrosis factor alpha
